# SATB2 Induces Malignant Transformation and Cancer Stem Cell Characteristics, and Inhibition of Its Expression Reverses Drug Resistance in Mesothelioma

**DOI:** 10.3390/cells15030283

**Published:** 2026-02-02

**Authors:** Cynthia Brown, Shivam Srivastava, Rohit Srivastava, Rashmi Srivastava, Jason Morvant, Anju Shrivastava, Rakesh K. Srivastava

**Affiliations:** 1 GLAX LLC, LSU Innovation Park, 8000 Innovation Park Drive, Baton Rouge, LA 70820, USA; cynthisbrown770@gmail.com (C.B.); shivamsri369@gmail.com (S.S.); rohitsri936@gmail.com (R.S.); rsssrs12@gmail.com (R.S.); 2 Department of Biological Sciences, Louisiana State University, Baton Rouge, LA 70803, USA; 3 Department of Chemistry and Biochemistry, Baylor University, Waco, TX 76706, USA; 4 Department of Surgery, Ochsner Health System, Gretna, LA 70056, USA; jasonmorvant@gmail.com; 5 St. Joseph’s Hospital and Medical Center, Phoenix, AZ 85013, USA; dras633@gmail.com

**Keywords:** mesothelioma, apoptosis, cancer stem cells, epithelial–mesenchymal transition, spheroids, Nanog, SATB2, drug resistance, transformation

## Abstract

**Highlights:**

**What are the main fundings?**

- SATB2 is highly expressed in mesothelioma.
- Inhibition of SATB2 expression reverses drug resistance and enhances the effects of chemotherapy in mesothelioma.

**What are the implications of the main findings?**

- SATB2 can serve as a diagnostic biomarker for mesothelioma.
- It can be considered a novel target for treating mesothelioma.

**Abstract:**

SATB2 (special AT-rich binding protein 2) functions as a chromatin-associated epigenetic regulator that modulates gene expression, in part by serving as a transcriptional cofactor. This study assessed whether SATB2 overexpression is sufficient to promote in vitro transformation of human mesothelial cells and whether SATB2 suppression in mesothelioma cancer stem cell (CSC)–enriched populations is associated with altered chemoresistance. SATB2 expression was high in human malignant pleural mesothelioma (MPM) cell lines but absent in Met5A mesothelial cells. Ectopic SATB2 expression in Met5A cells was associated with acquisition of malignant and stem cell–like phenotypes, including increased expression of stem cell markers and pluripotency-associated factors, as well as anchorage-independent growth in soft agar and spheroid formation in suspension culture. In contrast, Met5A cells transduced with an empty vector did not form colonies or mesospheres. SATB2 overexpression in Met5A cells was also associated with increased motility, migration, and invasion, accompanied by induction of epithelial–mesenchymal transition (EMT)–related transcription factors relative to empty vector controls. Conversely, shRNA-mediated SATB2 knockdown in an MPM cell line attenuated proliferation, EMT-associated features, and CSC-like characteristics. Chromatin immunoprecipitation assays identified SATB2 occupancy at promoter regions of Bcl2, XIAP, KLF4, c-Myc, NANOG, and SOX2, consistent with a role in transcriptional regulation of genes linked to transformation, pluripotency, cell survival, proliferation, and EMT. In CSC-enriched cells, SATB2 inhibition was associated with increased sensitivity to cisplatin and pemetrexed, concomitant with reduced OCT4 and SOX2 expression. Collectively, these findings support SATB2 as a candidate therapeutic target in MPM and suggest that SATB2 suppression may enhance chemotherapy response when combined with standard agents.

## 1. Introduction

Mesothelioma is a highly lethal malignancy associated with poor clinical outcomes [1,2,3,4,5,6]. The disease is primarily linked to asbestos exposure and, in a subset of cases, to exposure to the SV40 virus [7,8,9,10,11,12]. In the United States, approximately 2500 deaths from mesothelioma occur annually [13,14]. Malignant mesothelioma is an invasive cancer that arises predominantly from the mesothelial linings of the peritoneum, pleura, and pericardium. Among these anatomic sites, malignant pleural mesothelioma (MPM) is the most common presentation, accounting for approximately 70% of cases [4,6,15,16,17]. MPM is an aggressive disease that is associated with poor prognosis, late detection, metastasis, and resistance to therapy [4,6,9,18]. MPM is a disease of aging, as it primarily affects the elderly population [14]. Although a limited success has been made with the combination of pemetrexed and cisplatin (a standard care of therapy), the prognosis for mesothelioma patients is still very low, and median survival is between 12 and 17 months [19,20]. Mesothelioma imposes a substantial burden on patients’ quality of life across both physical and emotional domains and markedly affects families and caregivers. However, the molecular events underlying its development remain incompletely understood. Since there are no reliable biomarkers for detecting MPM, managing the disease becomes very challenging. Therefore, understanding the molecular mechanism by which normal mesothelial cells transform into malignant cells is of paramount importance.

SATB2 (special AT-rich binding protein-2) binds DNA at nuclear matrix attachment regions and modulates gene expression through effects on chromatin architecture and by acting as a transcriptional co-factor [21,22]. Structurally, SATB2 comprises five highly conserved domains, which are: (i) one ubiquitin-like domain (ULD), (ii) one CUT repeat-like (CUTL) domain, (iii) two CUT domains (CUT1 and CUT2), and (iv) one homeodomain (HOX) [23]. In addition to these domains, SATB2 contains two distinct nuclear matrix-attachment regions (MARs) and several sites for ubiquitin modifications [24]. As a nuclear matrix protein, it binds MARs, which are regulatory DNA sequences implicated in higher-order chromatin organization [25]. SATB2 uses its CUT domains to anchor to the sugar-phosphate backbone of DNA, providing a structural basis for chromatin loop formation and contributing to genome spatial organization. The establishment of anchored loops facilitates recruitment of coactivator and corepressor complexes, thereby tightly controlling gene expression and chromatin modifications [22]. In addition, it also functions as a scaffold protein, coordinating the assembly of additional DNA-binding factors at discrete subnuclear sites to support transcriptional regulation [26]. Consistent with roles in skeletal biology, SATB2−/− mice exhibit major defects in bone development and osteoblast differentiation [27]. Collectively, these observations support multifunctional roles for SATB2 in diverse biological processes, including skeletal formation, craniofacial patterning, osteoblast differentiation, nervous system development, and tumor progression [26,27,28].

Elevated SATB2 expression has been reported across multiple malignancies, including colorectal, pancreatic, breast, and prostate cancers, neuroendocrine tumors, and hepatocellular carcinoma, supporting its proposed utility as a cancer diagnostic marker [29,30,31,32,33,34,35,36,37]. In breast cancer, SATB2 mRNA levels show a significant association with higher tumor grade and reduced survival [37]. Increased SATB2 expression also correlates with immune cell infiltration [30]. In pancreatic cancer, SATB2 has been linked to suppression of T cell cytotoxicity together with enhanced tumor cell migration, suggesting that SATB2 may represent a target to improve pancreatic cancer immunotherapy. However, whether SATB2 contributes to tumor-promoting and metastatic phenotypes in mesothelioma has not been investigated.

Malignant transformation is a complex process in which a normal cell becomes cancerous. This transformation can occur through several mechanisms, including genetic mutations, changes in cellular regulatory mechanisms, the generation of reactive oxygen species, and the accumulation of mutations that confer a malignant phenotype. This transformation can generate cancer stem cells (CSCs) that express stem cell markers and pluripotency-maintaining factors. Our recent work has demonstrated that overexpression of the SATB2 gene induced malignant transformation in human breast, prostate, colorectal, and pancreatic epithelial cells and hepatocytes, resulting in the generation of functional CSCs [35,36,38,39,40]. These CSCs are considered to be responsible for cancer initiation, progression, drug resistance, and chemotherapy failure [41,42,43,44,45,46]. Although cell growth-enhancing and tumor-promoting roles of SATB2 in certain cancers have been demonstrated, its biological functions in the malignant transformation of mesothelial cells remain unexamined.

This study aimed to evaluate whether SATB2 expression is sufficient to promote cellular transformation in mesothelial cells and to assess whether suppressing SATB2 in MPM CSCs reverses drug resistance. The results showed that ectopic SATB2 expression in mesothelial cells was associated with malignant transformation, whereas shRNA-mediated SATB2 knockdown in MPM cells reduced cell growth and attenuated EMT, mesosphere formation, and CSC-associated properties. Knockdown of SATB2 expression in MPM CSCs by shRNA reversed drug-resistance to cisplatin and pemetrexed. SATB2 can be considered a therapeutic target in MPM, and inhibiting SATB2 in combination with chemotherapy may be a viable option for MPM treatment.

## 2. Materials and Methods

### 2.1. Reagents and Cell Culture Conditions

Antibodies against E-Cadherin, N-cadherin, SATB2, cMyc, Oct4, XIAP, and β-Actin were purchased from Cell Signaling Technology, Inc. (Danvers, MA, USA) and Abcam (Cambridge, MA, USA). Enhanced chemiluminescence (ECL) Western blot detection kit was purchased from Fisher Scientific (Pittsburgh, PA, USA). Human mesothelioma cell lines H2714, H2452, and H2595, and mesothelial cell line Met5A (CRL-9444) were purchased from American Type Culture Collection (ATCC), Manassas, VA, USA. H2741 cell line harbors mutations in BRCA2 (p.T3033NfsTer11 frameshift variant), Notch2 (p.A1083SfsTer17 frameshift variant), PIK3CA (p.Q546R missense variant), and TP53 (p.Q331R missense variant and p.V73WfsTer50 frameshift variant). H2452 cell line harbors mutations or gene deletion in BAP1 [Heterozygous mutation (p.Ala95Asp or c.284C>A), and gene deletion] and CDKN2A (Homozygous gene deletion). H2495 cell line harbors mutations in ARID1A (p.D1850GfsTer4 frameshift variant), ATM (p.K1101E missense variant), BRAF (p.L597R missense variant), BRCA2 (p.T3033NfsTer11 frameshift variant), NOTCH2 (p.A1083SfsTer17 frameshift variant), PIK3CA (p.Q546R missense variant), and TP53 (p.Q331R missense variant). The Met5A cell line was derived from human adult pleural mesothelial cells.

Mesothelioma cells were grown in Dulbecco’s Modified Eagle’s Medium supplemented with 10% fetal bovine serum (FBS) and 1% antibiotic-antimycotic solution. Met5A cells were grown in Medium 199 supplemented with FBS (10%), epidermal growth factor (10 ng/mL), hydrocortisone (400 nM), human recombinant insulin (870 nM), and 1% antibiotic-antimycotic solution. All the cells were grown in an incubator at 37 °C with 5% CO_2._

### 2.2. Lentiviral Particle Production and Transduction

Lentiviral particle generation in packaging cells and subsequent transduction of mesothelioma cells were carried out according to established procedures with minor modifications [47]. Briefly, 293T packaging cells were transfected with 4 µg plasmid DNA using a lipid-based method (Lipofectamine-2000/Plus reagent, Invitrogen, Carlsbad, CA, USA) in accordance with the manufacturer’s instructions. Following transfection, viral supernatants were harvested and concentrated by the addition of PEG-it virus precipitation solution (SBI System Biosciences, Mountain View, CA, USA) to generate virus stocks with titers of 1 × 10^8^ to 1 × 10^9^ infectious units per mL. Viral supernatant was collected by ultracentrifugation over three days and concentrated 100-fold. Met5A and mesothelioma cells were infected with lentiviral particles containing the gene of interest.

### 2.3. Immunofluorescence

Cells were seeded onto coverslips and cultured for 24 h, followed by fixation in 3.7% paraformaldehyde (Sigma-Aldrich, St. Louis, MO, USA) prepared in PBS for 10 min at room temperature. Fixed cells were then permeabilized with 0.5% Triton X-100 in PBS for 15 min, and subsequently blocked using 10% fetal calf serum in PBS supplemented with 0.1% Triton X-100 (Sigma). For SATB2 staining, cells were incubated overnight at 4 °C with an anti-SATB2 antibody (Abcam, Waltham, MA, USA), followed by 1 h at room temperature with the secondary antibody. Nuclear DNA was stained with DAPI (Thermo Fisher Scientific, Waltham, MA, USA). Fluorescent images were acquired using a fluorescent microscope with NIS-Elements software (Nikon, Tokyo, Japan).

### 2.4. Motility Assay

Horizontal cell movement was assessed using a scratch (wound-healing) motility assay performed as previously described [47]. Mesothelioma cells were seeded into 6-well cell culture plates and allowed to form a confluent monolayer. A linear scratch was then introduced to create an in vitro wound on a monolayer. After making a scratch, the plates containing cells were washed twice with PBS, and then fresh culture medium was added. Migration into the scratched area was subsequently monitored as individual cells advanced from the confluent margins. The width of the scratch gap was visualized under the Nikon microscope until the gap was filled in the untreated control wells.

### 2.5. Transwell Migration Assay

Mesothelioma cell migration was evaluated using a Transwell insert system as previously described [47]. Briefly, mesothelioma cells (1 × 10^5^) were seeded into the upper chamber of a 24-well insert (pore size, 8 μm; Corning, NY, USA) and permitted to migrate toward serum-containing medium placed in the lower chamber. Following 24 h of incubation, migrated cells were fixed in methanol and stained with 0.1% crystal-violet (Sigma-Aldrich, St. Louis, MO, USA). Cells that traversed the membrane were quantified by counting under a light microscope (Nikon).

### 2.6. Transwell Invasion Assay

Invasive capacity was assessed using a Matrigel-based Transwell assay as previously described [47]. In brief, cells (1 × 10^5^) were seeded into the upper chamber of a 24-well insert containing a Matrigel-coated membrane (pore size, 8 μM; Corning Costar). Cells were suspended in medium lacking serum and growth factors, whereas serum-supplemented medium was placed in the lower chamber to serve as a chemoattractant. After 48 h of incubation, non-invading cells remaining on the upper surface were removed with a cotton swab. Cells that traversed the pores and adhered to the lower membrane surface were fixed in methanol, stained with 0.1% crystal-violet, and quantified by counting five independent fields per well using a light microscope (Nikon).

### 2.7. Immunoblotting

Cells were lysed using RIPA buffer (Thermo Fisher Scientific) supplemented with protease and phosphatase inhibitor cocktails (Thermo Fisher Scientific). Crude proteins (40 µg) were resolved by SDS-PAGE, and transferred to PVDF membranes (Thermo Fisher Scientific). Membranes were then blocked in 5% dry milk prepared in Tris-buffered saline (TBS) at 37 °C for 2 h. After blocking, membranes were probed with primary antibody diluted in TBS (1:1000 dilution) overnight at 4° C with gentle shaking. After incubation overnight, the membranes were washed 3 times (15, 5, and 5 min) with TBS containing 0.05% Tween-20 (TBST) and incubated again with secondary antibody (1:3000–5000 dilution) for 45 min at room temperature (RT). After incubation with secondary antibody, membranes were washed with TBST three times (15, 5, and 5 min) at RT. Finally, proteins were visualized with the ECL detection kit (Thermo Fisher Scientific).

### 2.8. Immunohistochemical Staining

Normal mesothelial and mesothelioma tissues were purchased from tissuearray.com (Derwood, MD, USA). Tissue sections were incubated with anti-SATB2 antibody (1:100) at 4 °C overnight, then washed with PBS three times, and incubated with horseradish peroxidase (HRP) conjugated goat anti-mouse IgG polyclonal antibody for 30 min at RT. A goat anti-mouse HRP-polymer detection kit was used to stain the tissue slides. The staining intensity was graded as described elsewhere [35]. We have also plotted the receiver operating characteristic (ROC) curve.

### 2.9. Quantitative Real-Time PCR

Quantitative real-time PCR (qRT-PCR) assay was performed as we described elsewhere [38]. The primer sequences were published in our earlier papers [35,36,38,39].

### 2.10. Chromatin Immunoprecipitation (ChIP) Assay

Chromatin Immunoprecipitation (ChIP) assay was performed as we described elsewhere [38].

### 2.11. Statistical Analysis

Statistical significance between two groups was evaluated using an unpaired Student’s t-test. Comparisons involving more than two groups were analyzed by one-way analysis of variance (ANOVA). For the assessment of cell proliferation measured across multiple time points, two-way ANOVA was applied. Statistical analyses were performed using Prism 8 software (GraphPad Software, Inc., San Diego, CA). Data are reported as mean ± SD, and *p* < 0.05 was considered statistically significant.

## 3. Results

### 3.1. Differential Expression of SATB2 in Human Normal Mesothelial and Mesothelioma Cells/Tissues, and Survival Probability of Mesothelioma Patients

**Figure 1:**
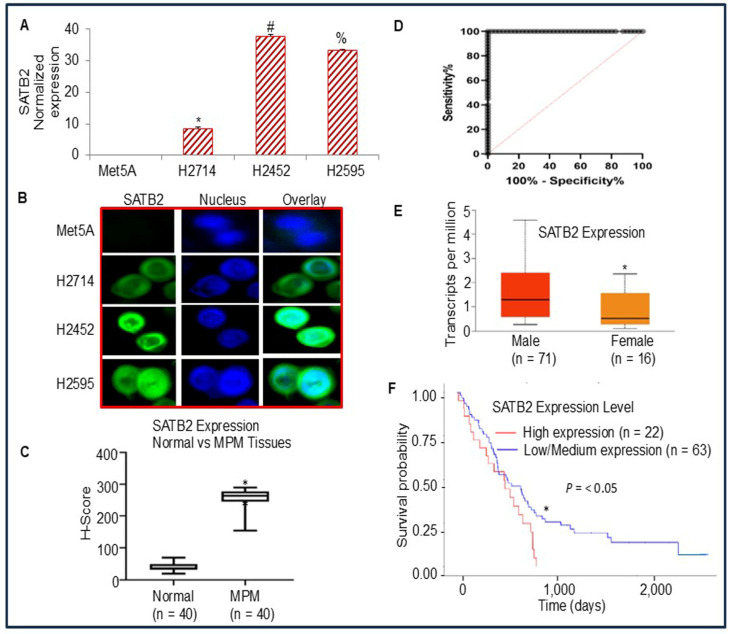
The expression of SATB2 in Met5A, and mesothelioma cell lines. (**A**), Expression of SATB2 mRNA in human normal mesothelial Met5A cells and mesothelioma cell lines. RNA was isolated, and qRT-PCR was used to measure SATB2 expression. *, #, and % = significantly different from Met5A (*p* < 0.05). (**B**), SATB2 expression by immunocytochemistry. Mesothelial and mesothelioma Met5A, H2714, H2452, and H2595 cells were grown in coverslips, fixed, and stained with anti-SATB2 antibody (Abcam, 1:500 dilution) for 24 h at 4 °C. Cells were then stained with secondary antibody and DAPI. Coverslips were mounted on the glass slides and imaged using a fluorescence microscope (Nikon). Green color = SATB2, Blue color = Nuclei. (**C**), H-Score of SATB2 protein expression in human normal mesothelial and mesothelioma tissues. Data represent mean (n = 40) ± SD. * = significantly different from normal (*p* < 0.001). (**D**), ROC Curve. ROC curve of normal and mesothelioma tissues. (**E**), SATB2 expression between males and females. TCGA data show a significant difference in SATB2 mRNA expression between samples from male (n = 71) and female (n = 16) mesothelioma patients (https://ualcan.path.uab.edu). Data represent mean ± SD. * = significantly different between males and females (*p* < 0.01). (**F**), TCGA data showing the effects of SATB2 expression on the survival probability curve of MPM patients expressing high and low/medium SATB2 levels. * = significantly different between the survival probability of MPM patients expressing high (n = 22) and low/medium (n = 63) SATB2 level (*p* < 0.05).

We first compared SATB2 expression in normal human mesothelial Met5A cells and mesothelioma cell lines (H2452 and H2595) by qRT-PCR. As shown in Figure 1A, SATB2 is not expressed in Met5A cells. However, it is highly expressed in mesothelioma cell lines. We next examined SATB2 expression by immunocytochemistry (Figure 1B). Met5A did not express SATB2. By comparison, SATB2 expression was observed in H2714, H2452, and H2595 cells. The lowest and highest SATB2 expression levels were observed in H2714 and H2452, respectively. These data suggest that SATB2 expression is tightly regulated in mesothelioma cells and may represent a novel therapeutic target.

We next compared the SATB2 expressions in human mesothelial and malignant pleural mesothelioma (MPM) tissues by immunohistochemistry (IHC) and quantified them by H-Score. As shown in Figure 1C, the expression of SATB2 was significantly higher in MPM tissues than in normal mesothelial tissues. According to the analysis of the ROC curve, SATB2 was expressed with high sensitivity and specificity (Figure 1D). Area under the ROC curve was 0.9939 ± 0.0005 SE (95% CI = 0.985 to 1, *p* < 0.0001). Because SATB2 is highly expressed in MPM tissues, patients are more likely to have aggressive disease than healthy individuals.

We next used the Cancer Genome Atlas (TCGA) data to examine whether SATB2 expression differs between male and female patients with MPM. SATB2 expression was significantly higher in male MPM patients than in females (Figure 1E).

Since SATB2 expression differs significantly between normal and mesothelioma patients, we next examined the effect of SATB2 expression on the survival probability of MPM patients using TCGA data. The survival curve showed that higher SATB2 expression was associated with poorer survival in MPM patients compared with those with low/medium SATB2 expression (Figure 1F). These data indicated that SATB2 expression was upregulated in MPM samples. Patients with higher SATB2 levels had a lower survival probability than those with low/medium SATB2 levels. Thus, SATB2 may serve as a specific prognostic biomarker in MPM patients.

### 3.2. Overexpression of SATB2 in Met5A Cells Induces Cellular Transformation

**Figure 2:**
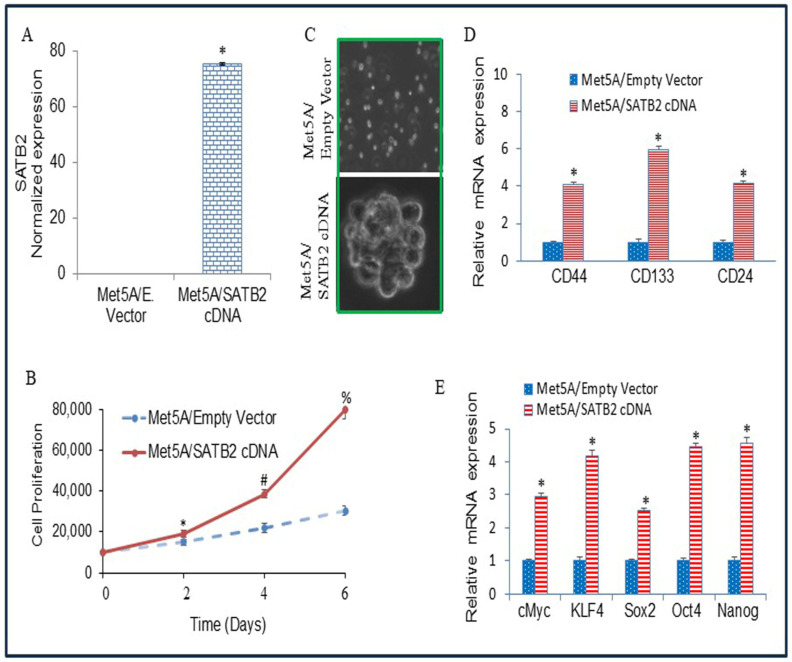
Overexpression of SATB2 in Met5A cells induces cellular transformation and stemness. (**A**), Met5A cells were stably transduced with lentiviral particles expressing either empty vector or SATB2 cDNA. QRT-PCR measured SATB2 expression. * = significantly different from each other, *p* < 0.05. (**B**), Cell proliferation. Met5A/Empty Vector and Met5A/SATB2 cDNA cells were grown, and cell proliferation was measured over 6 days. *, # and % = significantly different from respective empty vector group, *p* < 0.05. (**C**), Spheroid formation. Spheroid formation by Met5A/Empty Vector and Met5A/SATB2 cDNA cells in suspension was measured as we described elsewhere [36,38]. Mesospheres formed in 3 weeks were photographed. Cells transduced with an empty vector did not form any mesospheres. (**D**), RNA was isolated from Met5A/Empty Vector and Met5A/SATB2 cDNA cells. The expression of stem cell markers, CD44, CD133, and CD24, was measured by qRT-PCR analysis. * = significantly different from Met5A/Empty Vector group (*p* < 0.05). Gene expression of Empty Vector was normalized to 1. (**E**), RNA was isolated from Met5A/Empty Vector and Met5A/SATB2 cDNA cells. The expression of pluripotency-maintaining factors (cMyc, KLF4, Sox2, Oct4, and Nanog) was measured by qRT-PCR analysis. * = significantly different from Met5A/Empty Vector group (*p* < 0.05).

The cell transformation characteristics include uncontrolled and less oriented growth, the absence of contact inhibition, reduced dependence on growth factors, loss of tight junctions, and colony formation. To assess SATB2-associated transformation and stemness, SATB2 was overexpressed in normal human mesothelial Met5A cells. Lentiviral transduction generated Met5A/SATB2 cells with increased SATB2 mRNA expression (Figure 2A) and enhanced growth relative to Met5A/empty vector controls (Figure 2B). In suspension culture, Met5A/SATB2 cDNA cells formed spheroids (Figure 2C), whereas Met5A/empty vector cells did not. SATB2 overexpression was also associated with the induction of stem cell markers (CD44, CD133, and CD24) and transcription factors (cMyc, KLF4, Sox2, Oct4, and Nanog) (Figure 2D,E). Overall, these findings show that SATB2 overexpression in Met5A cells coincides with spheroid formation and increased expression of stemness-associated markers.

### 3.3. Overexpression of SATB2 in Met5A Cells Induces Epithelial-to-Mesenchymal Transition

**Figure 3:**
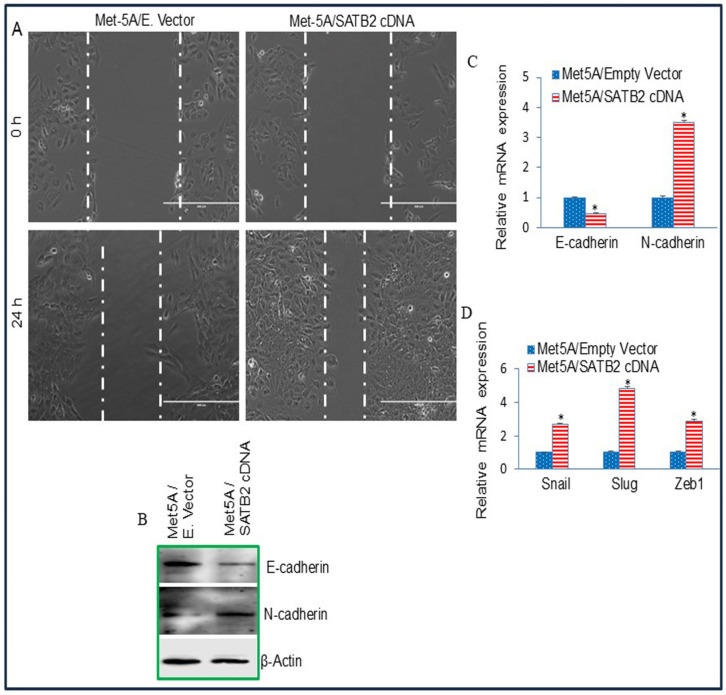
Overexpression of SATB2 in MET5A cells induces EMT characteristics. (**A**), Cell Motility assay. MET5A/Empty Vector and MET5A/SATB2 cDNA cells were grown in Petri dishes. After cell attachment, scratch lines were created using fine pipette tips in both groups. Phase-contrast images of scratched dishes were captured at 0 h and 24 h. (**B**), Protein expression of Cadherins. Western blot analysis was performed to measure the expression of E-cadherin and N-cadherin in MET5A/Empty Vector and MET5A/SATB2 cDNA cells. (**C**), mRNA expression of Cadherins in MET5A/Empty Vector and MET5A/SATB2 cDNA cells. qRT-PCR analysis was performed to measure the expression of E-cadherin and N-cadherin. * = significantly different from MET5A/Empty Vector (*p* < 0.05). Gene expression of the Empty Vector group was normalized to 1. (**D**), Expression of EMT-related transcription factors in MET5A/Empty Vector and MET5A/SATB2 cDNA cells. The expression of Snail, Slug, and Zeb1 was measured by qRT-PCR analysis. * = significantly different from MET5A/Empty Vector (*p* < 0.05).

Epithelial-to-mesenchymal transition (EMT) is a physiological process by which epithelial cells acquire a mesenchymal phenotype, thereby contributing directly to stemness and cancer cell metastasis [48,49]. We therefore assessed whether SATB2 overexpression in normal Met5A cells affects motility. As shown in Figure 3, Met5A mesothelial cells overexpressing SATB2 exhibited increased motility. EMT is accompanied by induction of EMT-related transcription factors and characteristic cadherin switching, with E-cadherin downregulated and N-cadherin upregulated [48,49]. Accordingly, E-cadherin and N-cadherin were compared between MET5A/Empty Vector and MET5A/SATB2 cDNA cells. Relative to MET5A/Empty Vector controls, MET5A/SATB2 cDNA cells showed reduced E-cadherin and increased N-cadherin at both the protein and mRNA levels. SATB2 overexpression also increased expression of the EMT transcription factors Slug, Snail, and Zeb1 in MET5A/SATB2 cDNA cells compared with MET5A/Empty Vector cells. These data suggest that SATB2 can induce EMT in MET5A cells.

### 3.4. Inhibition of SATB2 in Mesothelioma Cell Lines Results in Reduced Cell Proliferation, Decreased Colony Formation, and Decreased XIAP Expression

**Figure 4:**
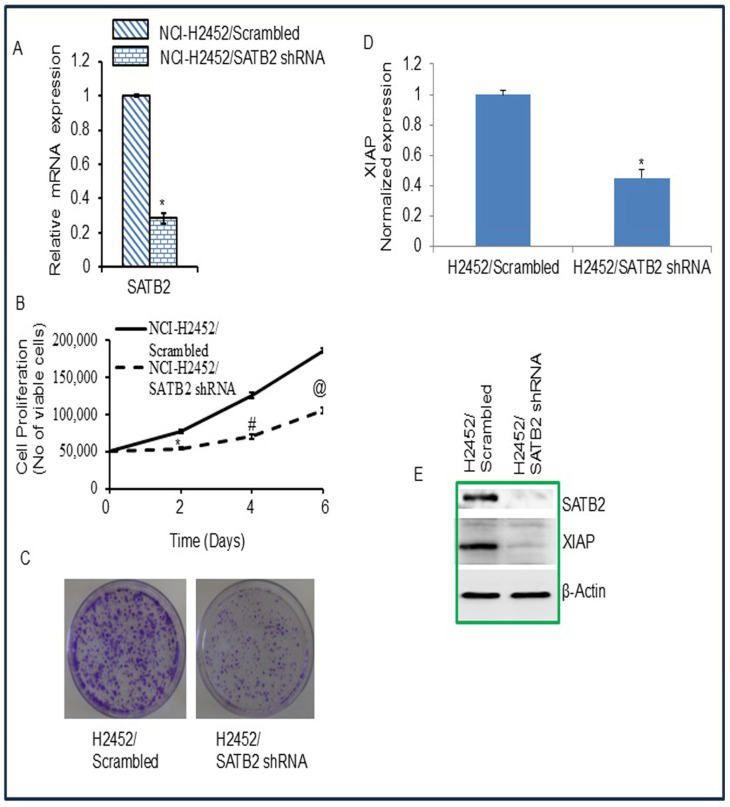
SATB2 shRNA inhibits cell proliferation, colony formation, and XIAP expression in H2452/Scrambled and H2452/SATB2 cells. (**A**), SATB2 expression. Mesothelioma H2452 cells were transduced with lentiviral particles expressing either scrambled or SATB2 shRNA (a mixture targeting four sites, Thermo Fisher). qRT-PCR was performed to measure SATB2 expression. * = significantly different from H2452/Scrambled group (*p* < 0.05). (**B**), Cell proliferation of H2452/Scrambled and H2452/SATB2 shRNA groups was measured over 6-day period. *, # and @ = significantly different from H2452/Scrambled group (*p* < 0.05). (**C**), Colony formation Assay. H2452/Scrambled and H2452/SATB2 shRNA cells were seeded, and colonies formed at 21 days were photographed. (**D**), Expression of XIAP mRNA. RNA was isolated from H2452 CSCs/Scrambled and H2452 CSCs/SATB2 shRNA cells. XIAP expression was measured by qRT-PCR. * = significantly different from H2452/Scrambled group (*p* < 0.05). (**E**), Protein expression of SATB2 and XIAP. Crude protein was isolated from H2452/Scrambled and H2452/SATB2 shRNA cells, and the Western blot analysis was performed to measure the expression of SATB2 and XIAP. β-actin was used as a loading control.

SATB2 is involved in maintaining stemness and promoting cell survival [35,36,38,39]. We next examined whether SATB2 inhibition attenuates the growth of the mesothelioma H2452 cell line. We used the H2452 mesothelioma cell line because it showed the highest SATB2 expression (Figure 1A). Knockdown of SATB2 by shRNA inhibited SATB2 expression, as measured by the qRT-PCR and western blot analysis (Figure 4A,E). H2452/SATB2 shRNA cells had a lower growth rate than H2452/Scrambled cells (Figure 4B). We next examined the effects of SATB2 on colony formation. SATB2 shRNA inhibited colony formation in H2452/SATB2 shRNA cells compared with the H2452/Scrambled group (Figure 4C). These data suggest that SATB2 knockdown in mesothelioma cells can suppress cell proliferation.

Since XIAP is a target of SATB2, we measured XIAP expression (mRNA and protein levels), which regulates cell proliferation. H2452/SATB2 shRNA group showed less expression of XIAP compared to that of H2452/scrambled group (Figure 4D,E). These data suggest that inhibition of SATB2 expression in mesothelioma cells can suppress cell proliferation and colony formation by modulating XIAP expression.

### 3.5. Inhibition of SATB2 in Mesothelioma Cell Lines Suppresses Epithelial–Mesenchymal Transition

**Figure 5:**
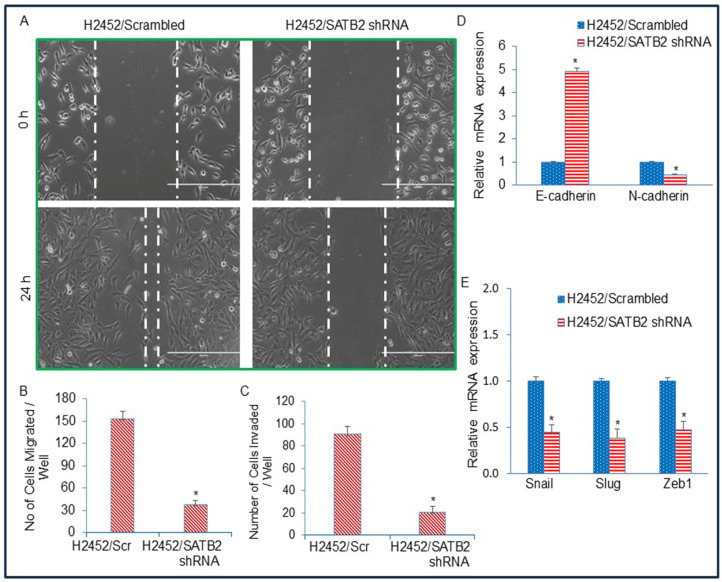
SATB2 shRNA inhibits cell motility, migration, and invasion and regulates EMT-related genes and transcription factors in H2452 cells. (**A**) Cell motility assay. H2452/Scrambled and H2452/SATB2 shRNA cells were cultured in Petri dishes. On the following day, linear scratches were introduced using fine pipette tips. After washing with PBS, fresh culture medium was added. Phase-contrast images of the wound area were acquired at 0 h and 24 h. (**B**) Transwell migration assay. H2452/Scrambled and H2452/SATB2 shRNA cells were analyzed using a Transwell migration assay as described in the Materials and Methods section. * = significantly different between groups (*p* < 0.05). (**C**), Transwell invasion assay. Invasive capacity of H2452/Scrambled and H2452/SATB2 shRNA cells was assessed using a Transwell invasion assay as described in the Materials and Methods. * = significantly different between groups (*p* < 0.05). (**D**), Expression of Cadherins. Total RNA was extracted from H2452/Scrambled and H2452/SATB2 shRNA cells, and E-cadherin and N-cadherin mRNA levels were quantified by qRT-PCR. * = significantly different between groups (*p* < 0.05). (**E**), RNA was isolated from H2452/Scrambled and H2452/SATB2 shRNA cells, and expression of Snail, Slug, and Zeb1 was determined by qRT-PCR. * = significantly different between groups (*p* < 0.05).

EMT is a biological process in which epithelial cells, which are typically structured, possess a defined shape and polarity, are tightly connected by junctions, and are stationary, transform into mesenchymal cells, which are more flexible, loosely coupled, highly mobile, and capable of migration. We next examined the effects of SATB2 inhibition on EMT and on the expression of EMT-related genes and transcription factors. As shown in Figure 5A, H2452/SATB2 shRNA cells demonstrated lower motility than those of H2452/Scrambled cells. Similarly, the H2452/SATB2 shRNA group exhibited lower migration and invasion than the H2452/Scrambled group (Figure 5B,C). We next examine the effects of STAB2 shRNA on Cadherin expression (Figure 5D). SATB2 shRNA upregulated the expression of E-cadherin and downregulated the expression of N-cadherin, a phenomenon known as “cadherin switch”, observed during EMT. Furthermore, SATB2 shRNA inhibited the expression of EMT-inducing transcription factors (Figure 5E). These data suggest that SATB2 inhibition can suppress EMT by modulating cadherin and EMT-related gene expression.

### 3.6. The Side Population of Malignant Mesothelioma Cells H2452 Contains CSCs, and Inhibition of SATB2 Suppresses Stemness in Mesothelioma

**Figure 6:**
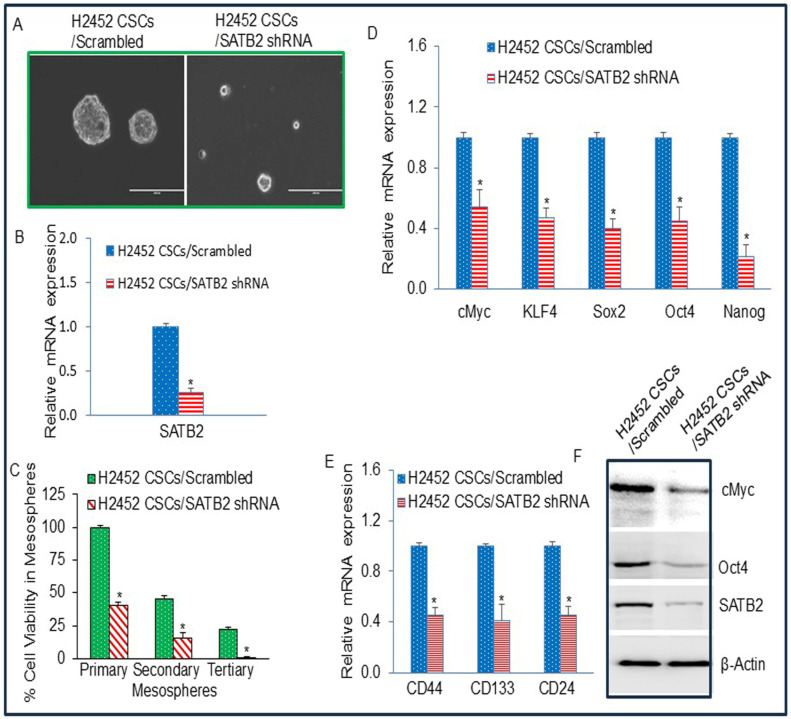
SATB2 shRNA inhibits spheroid formation, and the expression of stem cell markers and pluripotency factors in CSCs generated from H2452 cells. (**A**), SATB2 shRNA inhibits spheroid formation in CSCs (side populations) isolated from H2452 cells. H2452 cells were transduced with lentiviral particles expressing either Scrambled (H2452 CSCs/Scrambled) or SATB2 shRNA (H2452 CSCs/SATB2 shRNA). Cells were grown in an ultra-low attachment plate containing well-defined stem cell medium for 3 weeks. This process enabled us to select and grow the side population of N2452 cells, which exhibited stem-like cell properties. At the end of the incubation period, mesospheres were photographed. (**B**), Expression of SATB2. RNA was isolated from H2452 CSCs/Scrambled and H2452 CSCs/SATB2 shRNA cells. qRT-PCR was performed to measure SATB2 expression. * = significantly different from H2452 CSCs/Scrambled group (*p* < 0.05). (**C**), Cell Viability in Mesosphere. H2452 CSCs/Scrambled and H2452 CSCs/SATB2 shRNA cells were seeded on ultra-low attachment plate in suspension to obtain primary, secondary and tertiary mesospheres. Cell viability in mesosphere was measured by trypan blue assay at the end of 7, 14, and 21 days. * = significantly different from H2452 CSCs/Scrambled group (*p* < 0.05). (**D**), Expression of CSC markers CD44, CD133 and CD24. RNA was isolated from H2452 CSCs/Scrambled and H2452 CSCs/SATB2 shRNA cells. qRT-PCR analysis was performed to measure the expression of CSC markers CD44, CD133, and CD24. * = significantly different from H2452 CSCs/Scrambled group (*p* < 0.05). (**E**), Expression of pluripotency maintaining factors cMyc, KLF4, Sox2, Oct4, and Nanog. RNA was isolated from H2452 CSCs/Scrambled and H2452 CSCs/SATB2 shRNA cells. qRT-PCR analysis was performed to measure the expression of cMyc, KLF4, Sox2, Oct4, and Nanog. * = significantly different from H2452 CSCs/Scrambled group (*p* < 0.05). (**F**), Protein expression of cMyc, Oct4, and SATB2. Western blot analysis was performed to measure the expression of SATB2, cMyc, and Oct4 in H2452 CSCs/Scrambled and H2452 CSCs/SATB2 shRNA cells. β-actin was used as a loading control.

Cancer stem cells (CSCs) are a small subpopulation of cells within a tumor mass that behave like stem cells. They can self-renew and generate a diverse range of cell types. CSCs are capable of cancer initiation, progression, drug resistance, and chemotherapy failure (regenerating tumors after chemotherapy). They are highly mobile and adaptable. We therefore isolated a subpopulation of CSCs by growing them in low-attachment plates in stem cell medium. We allowed them to form mesospheres, a process well known for isolating the side population of CSCs. We next examined the effects of SATB2 shRNA on mesosphere formation by CSCs and on the expression of stem cell markers and pluripotency-maintaining factors. As shown in Figure 6A, the H2452/SATB2 shRNA group showed a larger mesosphere than the H2452/Scrambled group. Inhibition of SATB2 expression by shRNA inhibited SATB2 expression in H2452 CSCs (Figure 6B).

Since STAB2 shRNA inhibited MPM mesosphere formation, we next sought to examine the effects of inhibiting SATB2 expression by shRNA on cell viability of primary, secondary, and tertiary spheroids. Spheroid/mesosphere formation is a key characteristic and advantageous trait of cancer stem cells (CSCs), as these 3D structures more closely mimic the natural body environment than flat 2D cultures, thereby enhancing stemness, self-renewal, differentiating potential, and therapeutic properties. Tumor-derived spheroids (mesospheres) are used to isolate and study CSCs, which have been linked to tumor growth, recurrence, and chemoresistance. SATB2 shRNA significantly inhibited cell viability in primary, secondary, and tertiary mesospheres formed by H2452 CSCs (H2452 CSCs/SATB2 shRNA) compared to H2452 CSCs/Scrambled group (Figure 6C). These data suggest that SATB2 shRNA is capable of inhibiting stemness.

Since STAB2 shRNA inhibited mesosphere formation, we next examined its effects on the expression of stem cell markers and pluripotency-maintaining factors in mesospheres, which contain the CSC population. SATB2 shRNA inhibited the mRNA expression of stem cell markers (CD44, CD133, and CD24) and pluripotency-maintaining factors (cMyc, KLF4, Sox2, Oct4, and Nanog) (Figure 6D,E). Similarly, SATB2 shRNA inhibited the protein expression of cMyc, Oct4, and SATB2 (Figure 6F). These data suggest that inhibiting SATB2 expression can suppress the CSC population and that SATB2 shRNA can be combined with chemotherapy for the treatment of mesothelioma.

### 3.7. SATB2 Directly Binds to Bcl2, XIAP, KLF4, cMyc, Nanog, and Sox2 in H2452 CSCs

**Figure 7:**
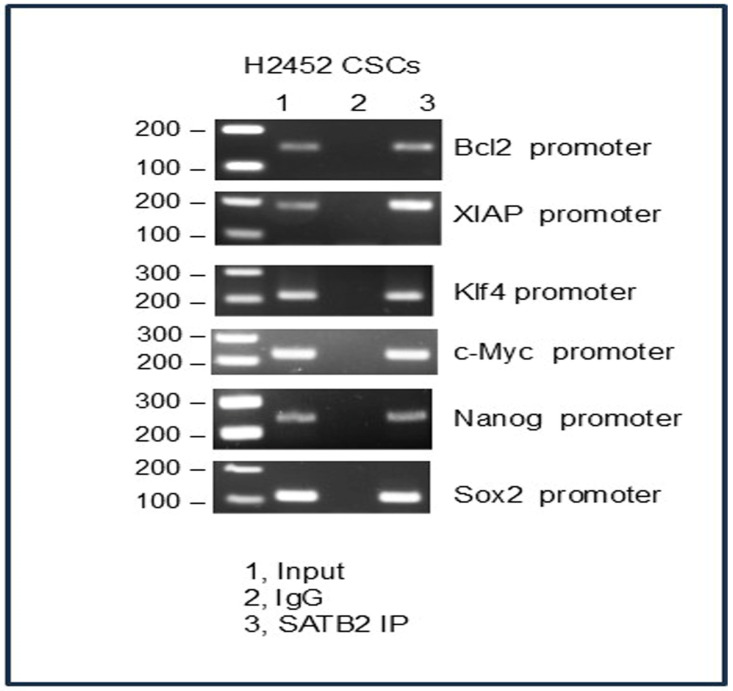
Binding of SATB2 to promoters of Bcl-2, XIAP, KLF4, cMyc, Nanog, and Sox2. Nuclear extracts were prepared from H2452 CSCs. Chromatin immunoprecipitation (ChIP) assays were performed as we described in Materials and Methods. The anti-SATB2 antibody (ab245424) used for immunoprecipitation was from Abcam (1:250 dilution). ChIP assays revealed SATB2 binding to the promoters of Bcl-2, XIAP, KLF4, cMyc, Nanog, and Sox2 in H2452 CSCs.

SATB2 is a transcription cofactor and chromatin modifier that regulates various biological processes such as stemness, cell proliferation, and survival [34,35,36,38,39,50]. Consistent with prior findings showing SATB2 occupancy at promoters of genes involved in stemness, cell growth, and survival [34,35,38,39,40], we used a chromatin immunoprecipitation (ChIP) assay to map SATB2-associated genomic regions. We next examined whether SATB2 directly binds Bcl2, XIAP, KLF4, cMyc, Nanog, and Sox2, which regulate stemness, cell growth, and survival, using ChIP assay in H2452 CSCs (Figure 7). SATB2 can directly bind to promoters of Bcl2, XIAP, KLF4, cMyc, Nanog, and Sox2. These results suggest that SATB2 may regulate multiple cellular functions through control of genes associated with stemness, cell proliferation, and survival.

### 3.8. The Inhibition of SATB2 Expression by shRNA Reverses Chemotherapy Resistance

**Figure 8:**
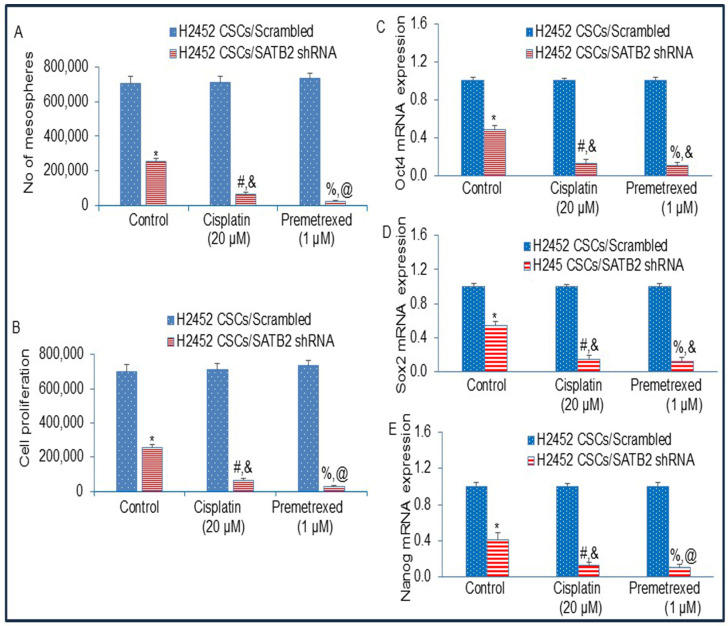
Inhibition of SATB2 expression in H2452 CSCs reverses drug-resistance to cisplatin and pemetrexed. (**A**), H2452 CSCs/Scrambled and H2452 CSCs/SATB2 shRNA cells were grown in an ultra-low attachment plate containing well-defined stem cell medium, and treated with or without Cisplatin (20 µM) or Premetrexed (1 μM) for 3 weeks. At the end of the incubation period, the numbers of mesospheres were counted. *. #, and % = significantly different from respective H2452 CSCs/Scrambled group (*p* < 0.05). *, & and @ = significantly different from each other (*p* < 0.05). (**B**), H2452 CSCs/Scrambled and H2452 CSCs/SATB2 shRNA cells were grown in an ultra-low attachment plate containing well-defined stem cell medium, and treated with or without Cisplatin (20 µM) or Premetrexed (1 μM) for 3 weeks. At the end of the incubation period, mesospheres were collected and dissociated with Accutase^TM^ (Stemcell Technologies, Kent, WA, USA). Viable cells were stained with typan blue (Invitrogen) and counted using a Countess Automated Cell Counter (Invitrogen). *. #, and % = significantly different from respective H2452 CSCs/Scrambled group (*p* < 0.05). *, & and @ = significantly different from each other (*p* < 0.05). (**C**), Expression of Oct4. H2452 CSCs/Scrambled and H2452 CSCs/SATB2 shRNA cells were grown in an ultra-low attachment plate containing well-defined stem cell medium, and treated with or without Cisplatin (20 µM) or Premetrexed (1 μM) for 3 weeks. RNA was isolated from H2452 CSCs/Scrambled and H2452 CSCs/SATB2 shRNA cells. qRT-PCR was performed to measure Oct4 expression. *. #, and % = significantly different from respective H2452 CSCs/Scrambled group (*p* < 0.05). *, and & = significantly different from each other (*p* < 0.05). (**D**), Expression of Sox2. H2452 CSCs/Scrambled and H2452 CSCs/SATB2 shRNA cells were grown in an ultra-low attachment plate containing well-defined stem cell medium, and treated with or without Cisplatin (20 µM) or Premetrexed (1 μM) for 3 weeks. RNA was isolated from H2452 CSCs/Scrambled and H2452 CSCs/SATB2 shRNA cells. qRT-PCR was performed to measure Sox2 expression. *. #, and % = significantly different from respective H2452 CSCs/Scrambled group (*p* < 0.05). *, and & = significantly different from each other (*p* < 0.05). (**E**), Expression of Nanog. H2452 CSCs/Scrambled and H2452 CSCs/SATB2 shRNA cells were cultured in ultra-low attachment plates in a well-defined stem cell medium and maintained for 3 weeks in the presence or absence of Cisplatin (20 µM) or Premetrexed (1 μM). Total RNA was then extracted from H2452 CSCs/Scrambled and H2452 CSCs/SATB2 shRNA cells, and Nanog expression was quantified by qRT-PCR. *, #, and % = significantly different from the corresponding H2452 CSCs/Scrambled group (*p* < 0.05). *, & and @ = significantly different from each other (*p* < 0.05).

MPM patients are generally treated with cisplatin and premetrexed, but patients suffer chemotherapy failure due to the generation of CSCs. Since CSCs are responsible for drug resistance and chemotherapy failure, we next sought to examine the effects of SATB2 shRNA on cisplatin- and premetrexed-resistance in CSCs isolated from H2452 cells. Cisplatin and premetrexed had no effect on mesosphere formation by CSCs (Figure 8A). In contrast, SATB2 shRNA inhibited mesosphere formation. Furthermore, inhibition of SATB2 expression by shRNA sensitized CSCs to Cisplatin and premetrexed treatment.

We examined the cell viability in the mesosphere formed after drug treatment. Cisplatin or premetrexed did not affect mesospheres’ cell viability. SATB2 shRNA inhibited cell viability in the mesosphere compared to the scrambled group. Furthermore, SATB2 shRNA sensitized CSCs to Cisplatin or premetrexed treatment. These data suggest that (i) Induction of drug sensitivity by SATB2 shRNA resulted in a reduction in mesosphere formation and cell viability, and (ii) CSCs confer drug-resistance to cisplatin and premetrexed, and SATB2 shRNA sensitized CSCs.

## 4. Discussion

In the current study, we have demonstrated that overexpression of SATB2 gene alone can induce oncogenic transformation in human mesothelial cells. These transformed cells acquired CSC phenotypes, as they express pluripotency-maintaining factors and stem cell markers. These SATB2-transformed cells formed mesospheres in suspension, a characteristic of CSCs. Also, they acquired EMT characteristics, as evidenced by down-regulation of E-cadherin and up-regulation of N-cadherin, and by enhanced cell motility, migration, and invasion. Inhibition of SATB2 with shRNA sensitized CSCs to cisplatin and premetrexed. Overall, our study suggests that the SATB2 gene alone can induce oncogenic transformation of mesothelial cells, and inhibition of its expression could be beneficial for reversing drug resistance in MPM.

SATB2 has been proposed as a potential therapeutic target in several malignancies. We previously reported that SATB2 overexpression in normal human pancreatic, breast, colorectal, and prostate epithelial cells, as well as hepatocytes, is associated with malignant transformation accompanied by generation of CSCs [35,36,38,39,51]. In the current study, SATB2 overexpression likewise promoted transformation of Met5A cells, and the resulting cells displayed CSC-like features comparable to those described in humans and mice [52]. In addition, SATB2 was not detected in Met5A cells, whereas it was highly expressed across human mesothelioma cell lines. Consistent with these observations, shRNA-mediated suppression of SATB2 in mesothelioma cells reduced proliferation, colony formation, motility, migration, and invasion. SATB2 is known to cooperate with other transcription factors to modulate gene expression [53]. In tumors, SATB2 has been considered a biomarker and a candidate therapeutic target based on its involvement in regulation of oncogenic and tumor-suppressor pathways [54]. Our ChIP assays demonstrate that SATB2 directly binds the promoters of Bcl2, Xiap, Bsp, Klf4, Myc, Hoxa2, and Nanog, which play significant roles in cell proliferation, differentiation, EMT, and stemness. Overall, our data suggest that SATB2 can promote EMT and metastasis.

Work in solid organ malignancies indicates that CSCs represent a distinct subset of malignant precursors implicated in tumor initiation and progression and associated with resistance to cytotoxic agents, recurrence, and metastatic spread [55]. In malignant peritoneal mesothelioma, tumors have been reported to contain stem cells with tumorigenic capacity, a finding that informs considerations of the cells of origin and disease evolution in MPM [46]. The side population of malignant mesothelioma cells contains CSCs that express CD9, CD24, and CD26, which could serve as novel therapeutic targets [43]. The OCT4/SOX2 reporter method was used to identify CSC-enriched MM cell subpopulations that were resistant to cisplatin [56]. In a separate study, MPM cells resistant to combined cisplatin and the aldehyde dehydrogenase (ALDH) inhibitor diethylaminobenzaldehyde (DEAB) exhibited increased mRNA expression of ALDH1A2, ALDH1A3, and CD44, consistent with a role for these markers in chemoresistance [57]. Similarly, we showed that MPM cells can be enriched for CSCs that expressed stem cell markers and pluripotency-maintaining factors. Furthermore, induction of drug sensitivity by SATB2 shRNA resulted in reduced mesosphere formation and cell viability. Together, our findings support the presence of putative CSCs associated with resistance to cisplatin and pemetrexed in MPM. Targeting these drug-resistant MPM CSCs with SATB2 shRNA may enable more focused and effective chemotherapeutic regimens for MPM.

This study has some limitations. First, the experiments were carried out in vitro. Future studies will be needed to examine the oncogenic role of SATB2 in vivo. Second, we have used one normal mesothelial cell line, Met5A, in which SATB2 was overexpressed. Using another cell line will increase the study’s robustness. Third, the Met5A cell line was immortalized with SV40 large T-antigen, which may have influenced the study results. However, using human normal epithelial cell lines from the breast, prostate, colon, pancreas, and liver, we demonstrated that overexpression of the SATB2 gene induced oncogenic transformation, suggesting an oncogenic role for SATB2 [35,36,38,39,40]. Fourth, the therapeutic potential of SATB2 can be further evaluated by developing a novel small-molecule inhibitor for mesothelioma treatment.

In conclusion, our data suggest that SATB2 can induce oncogenic transformation of normal mesothelial cells into cancer stem-like cells. SATB2 can regulate several cellular functions by modulating the expression of genes involved in malignant transformation, pluripotency, cell survival, proliferation, and EMT. Induction of drug sensitivity by SATB2 shRNA resulted in reduced mesosphere formation and cell viability, and CSCs confer cisplatin- and premetrexed-resistance. Overall, targeting CSCs may be a valuable strategy to inhibit oncogenic progression. SATB2 can be considered as a therapeutic target in MPM, and inhibiting it in combination with chemotherapy may be a viable treatment option.

## Data Availability

The raw data supporting the conclusions of this article will be made available by the authors on request.
